# Influence of the adsorption geometry of PTCDA on Ag(111) on the tip–molecule forces in non-contact atomic force microscopy

**DOI:** 10.3762/bjnano.5.9

**Published:** 2014-01-27

**Authors:** Gernot Langewisch, Jens Falter, André Schirmeisen, Harald Fuchs

**Affiliations:** 1 Physikalisches Institut, Westfälische Wilhelms-Universität Münster, Wilhelm-Klemm-Straße 10, 48149 Münster, Germany; 2 Center for Nanotechnology (CeNTech), Westfälische Wilhelms-Universität Münster, Heisenbergstraße 11, 48149 Münster, Germany; 3 Institut für Angewandte Physik, Justus-Liebig-Universität Gießen, Heinrich-Buff-Ring 16, 35392 Gießen, Germany

**Keywords:** atomic force microscopy, organic molecules, three-dimensional (3D) force spectroscopy

## Abstract

Perylene-3,4,9,10-tetracarboxylic dianhydride (PTCDA) adsorbed on a metal surface is a prototypical organic–anorganic interface. In the past, scanning tunneling microscopy and scanning tunneling spectroscopy studies of PTCDA adsorbed on Ag(111) have revealed differences in the electronic structure of the molecules depending on their adsorption geometry. In the work presented here, high-resolution 3D force spectroscopy measurements at cryogenic temperatures were performed on a surface area that contained a complete PTCDA unit cell with the two possible geometries. At small tip-molecule separations, deviations in the tip-sample forces were found between the two molecule orientations. These deviations can be explained by a different electron density in both cases. This result demonstrates the capability of 3D force spectroscopy to detect even small effects in the electronic properties of organic adsorbates.

## Introduction

Perylene-3,4,9,10-tetracarboxylic dianhydride (PTCDA) adsorbed on the Ag(111) surface is a prototypical organic–anorganic interface that has been investigated by a large variety of different methods in the past [1]. Based on scanning tunneling microscopy (STM) and scanning tunneling spectroscopy (STS) experiments as well as theoretical simulations, it was found that the differences between the two possible adsorption geometries of PTCDA on the Ag(111) substrate affect the electronic structure of the molecules [2–5]. The chemical nature of the molecule–substrate bond leads to a charge transfer from the metal surface into the former LUMO of the molecules, however to a different extend for the two cases. As a result, the energetic centers of the now partially occupied LUMO levels are located at energies below the Fermi level with a shift of about 160 meV between the two adsorption geometries [4]. These deviations in the spectral weight below the Fermi level correspond to different electron densities.

Atomic force microscopy (AFM) investigations indicate that electronic properties such as the electron density distribution or partial charges within organic adsorbates are reflected by the tip–molecule force interactions [6–9]. However, a correlation between the electronic differences of the two PTCDA molecules of the unit cell and a force contrast in AFM experiments has not been reported yet. A possible reason might be the fact that the tip–sample forces are a complex superposition of several contributions of different physical effects and therefore of different range. AFM topography scans provide only a cut through the three-dimensional force field at a certain tip–sample separation. If the relevant interactions happen at a different distance, such an effect would not be detected. Therefore, we used the method of high-resolution three-dimensional (3D) force spectroscopy [10] to investigate the complete force field quantitatively, while starting at large tip–sample distances with no force interactions down to the regime of repulsive forces.

## Experimental

The experiments have been performed with a commercial low-temperature atomic force microscope (Omicron LT-SPM) that was operated in frequency-modulation mode [11] under ultrahigh vacuum conditions and at a temperature of ≈5 K using a tuning fork sensor (resonance frequency *f*_0_ = 24640 Hz, spring constant *k* ≈ 2000 N/m) in the qPlus design [12]. The amplitude of the sensor oscillation was held constant at *A* = 0.40 nm. To avoid crosstalk between tunneling current and deflection signal, no voltage was applied to the tip during NC-AFM operation. The tip was prepared by voltage pulses and soft indentation into the Ag sample most likely resulting in a Ag-terminated apex. However, it can not be excluded that a PTCDA molecule was picked-up afterwards during the scans. The PTCDA molecules were evaporated from a Knudsen cell up to a submonolayer coverage onto a clean Ag(111) surface, which was kept at room temperature during the deposition. More experimental details and previous measurements on the same system have been published before [3,13].

**Figure 1:**
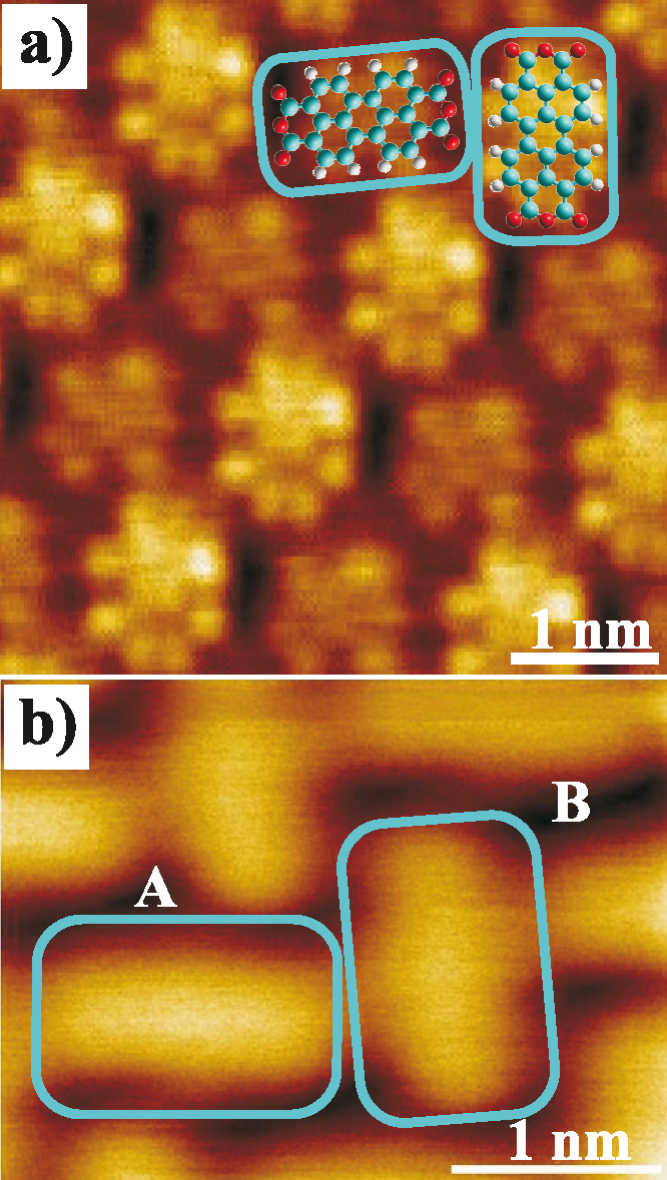
(a) STM topography scan (*V* = 0.2 V, *I* = 0.3 nA) of PTCDA on Ag(111). The two molecule orientations (turquois rectangles) of the unit cell correspond to different adsorption geometries and are imaged with a different intensity. (b) NC-AFM topography scan (Δ*f* = −0.6 Hz) of the surface area where the 3D force spectroscopy measurement was performed. No difference between the two orientations is detectable.

Figure 1a shows an STM topography image of a PTCDA monolayer on Ag(111). The molecules are arranged in a characteristic herring bone structure where the unit cell contains two molecules with different orientation and adsorption geometry. Here, a significant difference in the intensity of the two adsorption geometries can be observed, which is caused by the different electronic structure as described above. In contrast, this effect is not detectable in the AFM topography image in Figure 1b. However, as discussed above, the reason might be that the interactions correlated to this effect are not present at the tip–sample distance at which this scan was recorded (at a relatively large distance). Thus, 3D force field spectroscopy as described by Hölscher et al. [14] was utilized for a detailed investigation of the tip–molecule interactions. Above the surface area shown in Figure 1b, the frequency shift Δ*f*(*x*,*y*,*z*) was measured with 40 by 30 by 200 data points within a volume of 3.2 by 2.4 by 1.0 nm. In order to account for interactions that are not site-specific and beyond the *z* range, which was covered by this measurement, a separate Δ*f*(*z*) curve was recorded and added to the Δ*f*(*x*,*y*,*z*) matrix, by this expanding the *z* range to 10.2 nm in total. The resulting dataset was then converted into a three-dimensional landscape of vertical tip–sample forces BY using the Sader–Jarvis-algorithm [15]. As the duration of the 3D force spectroscopy measurement was about 5 3/4 h, the lateral drift of ≈40 pm/h led to a distortion of the originally rectangular surface area. In addition, a continuous drift of the frequency shift reference point of the order of 0.1 Hz/h was observed. The precise drift as a function of time was determined by a comparison of the Δ*f*(*z*) curves in the distance regime, in which no site-specific variations appeared (at large *z* distances) and used for a correction of the Δ*f*(*x*,*y*,*z*) matrix.

## Results and Discussion

**Figure 2:**
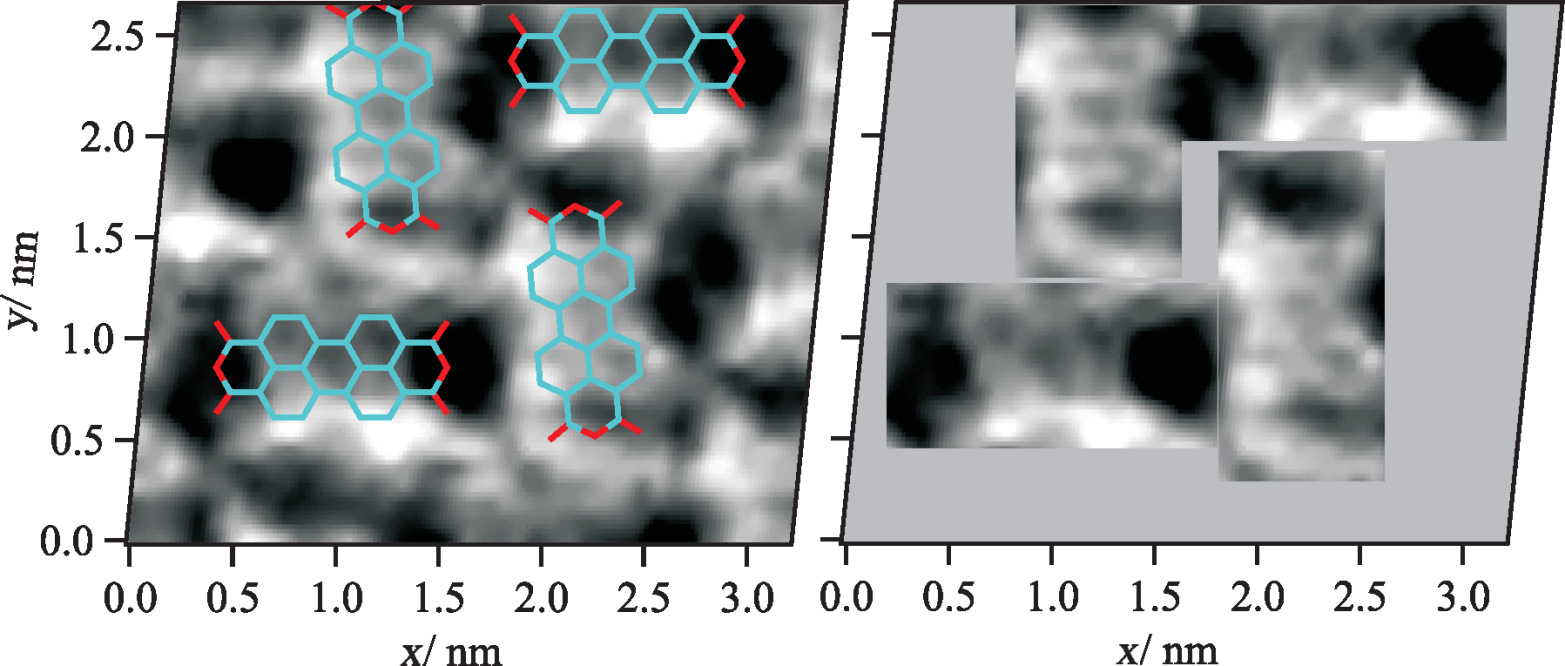
Horizontal cut through the 3D field of the vertical tip–sample forces at a distance of *z* = 0.60 nm (left). Dark features correspond to areas of enhanced attractive forces (for a quantitative analysis: see below in Figure 3 and Figure 4). The lateral drift was corrected in the images resulting in a distortion of the originally rectangular surface area. The images were linearly interpolated with a factor of 4 to enhance the visibility. Intramolecular structures can be seen in both the raw data images and the interpolated images. The characteristic shape of the perylene core consisting of five carbon rings can be identified, which allows an exact determination of the molecule positions in the 3D force spectroscopy measurement. The areas that can be assigned to PTCDA molecules are shown separately (right).

Figure 2 shows a horizontal cut through the 3D landscape of the vertical tip–sample forces at a distance of *z* = 0.60 nm. Please note that the origin of the *z* axis was defined arbitrarily as the absolute distance was unknown in the experiment. In this cut, intramolecular structures can be observed that can unambiguously be assigned to specific parts of the molecules. In particular, the characteristic structure of the five carbon rings in the perylene core can be identified. As already described by Moll et al. for PTCDA on Cu(111) [16], the contrast in AFM images recorded at tip–sample distances that correspond to the regime of repulsive forces reflects the electron density distribution of the molecules. This effect allows for a precise localization of the molecules and molecule moieties.

**Figure 3:**
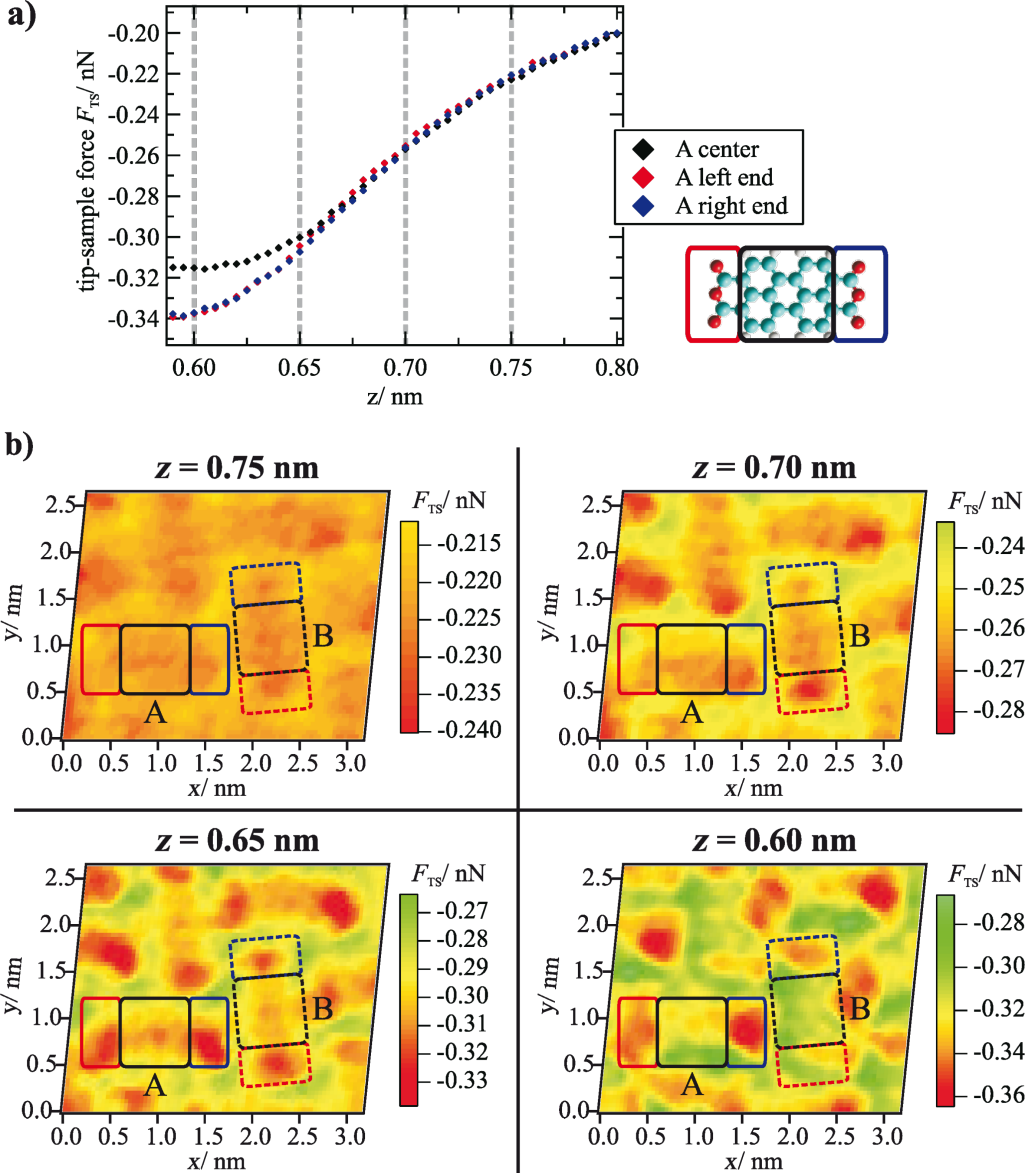
(a) Tip–sample forces as a function of *z* distance averaged for the area above the end groups and the center of a molecule (here: orientation A) as indicated by colored rectangles in the sketch on the right side. (b) Horizontal cuts through the 3D force field at different *z* distances that are marked by gray dashed lines in the *F*_TS_(*z*) curves in (a). In the cuts at *z* = 0.65 nm and *z* = 0.60 nm, a clear intramolecular contrast can be observed. In addition, at *z* = 0.60 nm, differences between the two molecule orientations appear. For all cuts, the color-force gradient is the same. However, the minimum of the color scale was adjusted to match the minimum force value within the cut. Furthermore, a slight linear interpolation with a factor of 2 was applied.

To analyze the general evolution of the tip–sample forces as a function of the *z* distance as well as site-specific effects, *F*_TS_(*z*) curves were averaged for the different molecule moieties (see sketch in Figure 3a: colored rectangular areas; number of curves used for the averaging was 5 × 8 per small rectangle and 8 × 8 per large rectangle). The resulting curves are shown exemplarily for the orientation A in Figure 3a. In this graph, the minimum of the *z* axis is the lower limit of the *z* range covered by the 3D force spectroscopy measurement. The evolution of the forces up to this point is typical and basically reflects a superposition of attractive long range (and additional attractive short range) forces with repulsive short range forces. When extrapolating this progression toward smaller distances, it can be assumed that the minimum of the *F*_TS_(*z*) curves is reached at *z* ≈ 0.60 nm. The net force is attractive at this point and about −0.32 ± 0.06 nN on average. As this distance is below the turning point of the curve, which is at *z* ≈ 0.67 nm, the absolute value of the force gradient is declining with decreasing *z*. This means that repulsive forces are acting and are partially compensating the attractive forces in this distance regime.

When comparing the interactions at the different molecule moieties, a general trend can be observed, which is similar for both orientations of the unit cell. While at large distances only small deviations between the end groups and the center are detectable, the differences are significant at distances of 0.65 nm and below. Here, the interactions at the end groups are more attractive. This trend is also obvious in horizontal cuts through the 3D force field at different distances (Figure 3b). At *z* = 0.75 nm, the molecules appear as featureless ovals. With decreasing distance, intramolecular structures arise that are clearly visible at distances of *z* ≤ 0.65 nm with areas of enhanced attractive forces (depicted in red) at the end groups.

**Figure 4:**
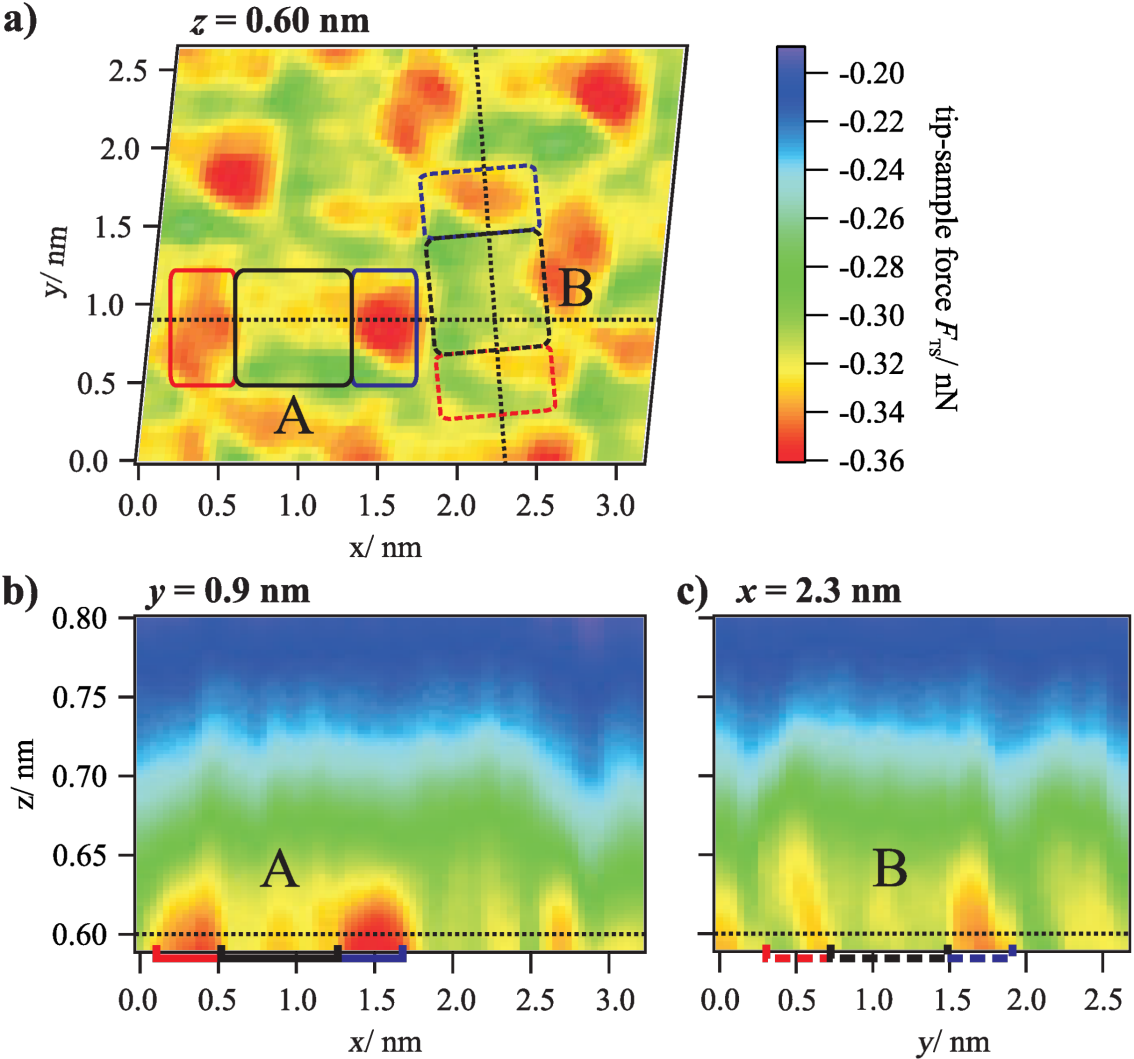
(a) Horizontal cut through the 3D force field at *z* = 0.60 nm and (b) vertical cuts at *y* = 0.9 nm and *x* ≈ 2.3 nm (diagonal progression) along the long axis of two different molecule orientations of the unit cell (the images are linearly interpolated with a factor of 2). For *z* < 0.65 nm, higher attractive forces are acting on molecule A in comparison to molecule B.

However, more interesting than this common trend are the differences between the two molecular orientations A and B. While in the horizontal cuts at *z* = 0.75 nm, 0.70 nm and 0.65 nm the two molecules of the unit cell appear nearly the same, at small distances of *z* = 0.60 nm, higher attractive tip–sample forces are acting on molecules with orientation A. To illustrate this effect in more detail, vertical cuts through the 3D force field are shown in Figure 4b and Figure 4c. These cuts run along the long axis of both molecule orientations as indicated by the dashed lines in Figure 4a. Here, the behavior observed in the horizontal cut at *z* = 0.60 nm can be found again: All partial groups of molecule A exert higher forces on the tip than the corresponding groups of molecule B. The onset of this trend is in the distance range between 0.65 and 0.60 nm. Additionally we find an asymmetry of the forces above the two ends on the molecules, for molecule A as well as for molecule B. Previous ab-initio simulations of PTCDA on Ag(111) predict a slight asymmetry of the end groups in the dissipation channel at small distances [17], but only for one molecular orientation. Therefore we speculate that this effect is related to an asymmetry of the tip apex in this experiment [18].

**Figure 5:**
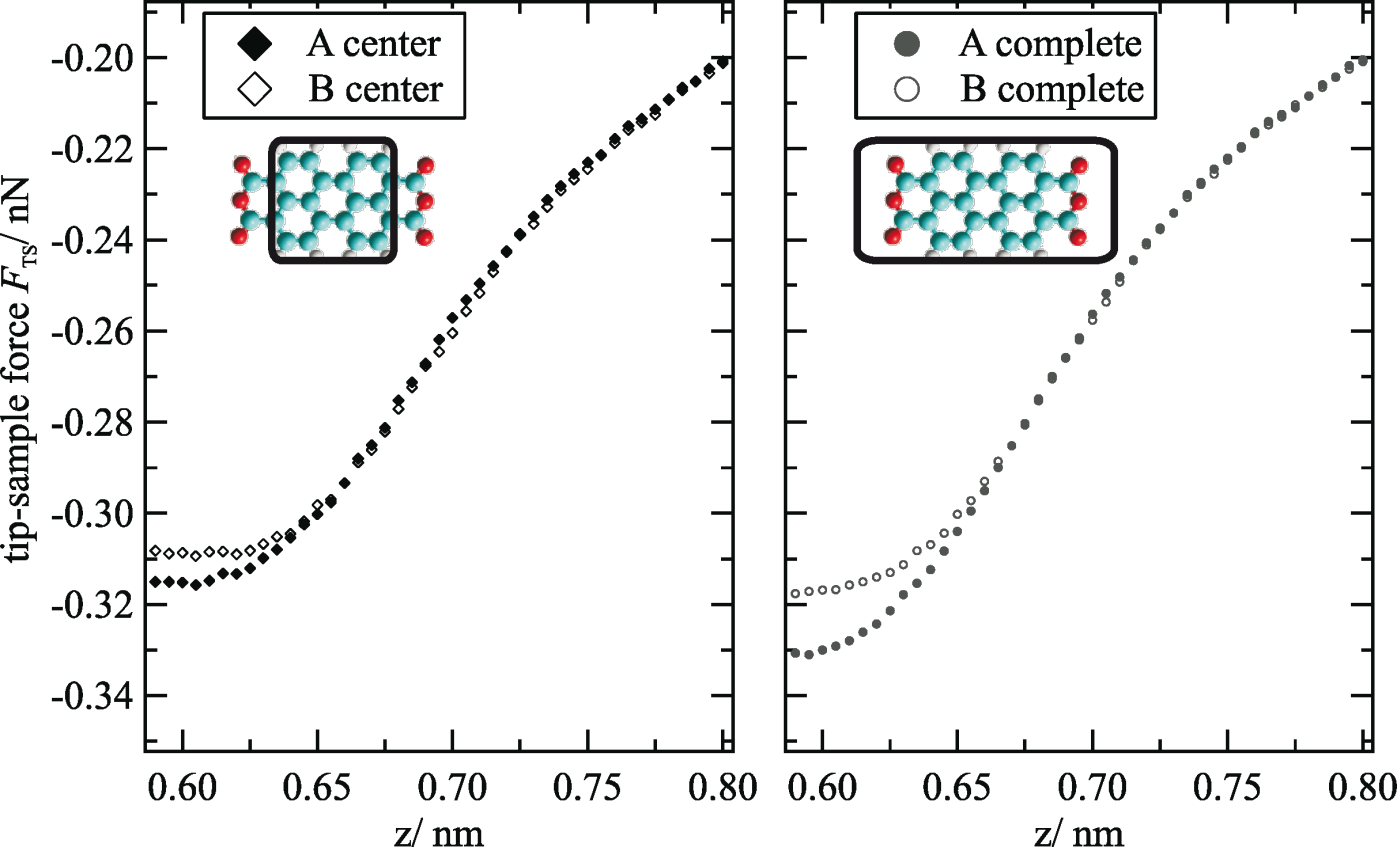
Comparison of force versus distance curves for molecule sites A and B. The forces are averaged for the perylene core (left) and the complete molecule (right) as indicated in the sketches of the molecules. At *z* distances below 0.65 nm, higher attractive forces are acting at molecule A.

This onset can be determined more accurately based on a comparison of the *F*_TS_(*z*) curves for both molecule orientations. The corresponding curves, which are averaged for the perylene cores (left) and the complete molecules (right), are shown in Figure 5. The deviations between the two orientations start obviously at *z* = 0.65 nm. At *z* = 0.60 nm, the difference, which is slightly lower at the perylene core in comparison to the complete molecule, is about 0.01 nN.

When analyzing the qualitative behavior of the tip–sample forces for the two molecule orientations individually, the evolution of the intramolecular contrast as a function of the distance *z* is as expected. At larger distances, in the regime of attractive long-range interactions such as van-der-Waals forces, no internal structures can be observed in the horizontal cuts through the 3D force field. The molecules appear as featureless ovals, which results in a corresponding contrast in the topography images recorded at small frequency shift values and, thus, large tip–sample distances (compare Figure 1b). Below a certain distance, the regime of short-range forces such as chemical interactions, short-range electrostatic forces or Pauli repulsion is reached. These forces most likely lead to the barbell-like structure as shown in Figure 3b at *z* = 0.65 nm with higher attractive forces at the end groups. While the physical origin of this contrast is not clear, a likely explanation for the structure that illustrated in Figure 2, in which the five carbon rings of the perylene core are visible, is provided by Gross and Moll et al. [8,16]. As was found for the first time for pentacene molecules and later also for PTCDA adsorbed on Cu(111), the atomic contrast revealing the carbon rings of organic molecules can be assigned to Pauli repulsion and reflects the electron density distribution of the molecules. Therefore, it can be assumed that Pauli repulsion also represents a significant contribution to the force interactions at small tip–sample distances in our measurements. Here, the characteristic contrast appears at distances below *z* ≈ 0.65 nm, at first only weakly and then increasing. As one would expect, this distance regime starts near the turning point of the *F*_TS_(*z*) curves, the point at which repulsive forces begin to compensate the attractive interactions.

Interestingly, this distance of about 0.65 nm is also the point at which the differences between the two molecule orientations arise. Thus, one can suspect that both effects have a common origin, i.e., that the different tip–sample forces are also related to Pauli repulsion. Deviations of this force interaction, which is sensitive to the electron density, could in this case be explained by the different spectral weight of the energetically shifted LUMO state (the hybrid state resulting from the chemisorption) below the Fermi level and corresponding differences in the electron density of the two orientations. A higher electron density would lead to a stronger repulsion and, thus, less attractive net forces near the minimum of the *F*_TS_(*z*) curves. Please note that no STM measurements were performed on the surface area investigated by the 3D force spectroscopy. Therefore, it is not possible to unambiguously verify this proposed relationship between electronic properties and forces in this case. However, the fact that the deviations in the tip–sample forces for the two orientations can be observed at the perylene core as well as at the end groups is in agreement with this interpretation because the LUMO state extends over the entire molecule.

As this effect can only be observed in the regime of repulsive forces near the minimum of the *F*_TS_(*z*) curves, it is not surprising that it was not observed in NC-AFM topography scans, yet. Such scans are in most cases recorded at a constant frequency shift. In the repulsive force regime (in which the repulsive forces start to partially compensate the attractive forces), the non-monotonic behavior of the frequency shift as a function of the tip–sample distance makes a stable operation of the distance feedback loop impossible. Furthermore, a stable and inert tip is required to avoid that the tip deforms or picks up the molecule. This is an additional factor that complicates AFM measurements at small tip–sample separations.

## Conclusion

Our 3D force spectroscopy measurements allow for a quantitative determination of the forces between an AFM tip and a PTCDA molecule on a Ag(111) surface as well as a detailed analysis of the qualitative evolution of the forces in three dimensions with submolecular resolution. In the regime of repulsive forces, a clear difference in the tip–sample forces was found between the two molecule orientations of the unit cell. For one orientation, the net force is higher than for the other one, an effect that extends over the complete molecule. This observation can be explained by the different electronic structure of the two orientations and demonstrates the capability of high-resolution 3D force spectroscopy to detect even minor deviations in the electronic properties of organic adsorbates.

## References

[1] Tautz F S (2007). Prog Surf Sci.

[2] Umbach E, Glöckler K, Sokolowski M (1998). Surf Sci.

[3] Braun D-A, Schirmeisen A, Fuchs H (2005). Surf Sci.

[4] Kraft A, Temirov R, Henze S K M, Soubatch S, Rohlfing M, Tautz F S (2006). Phys Rev B: Condens Matter Mater Phys.

[5] Rohlfing M, Temirov R, Tautz F S (2007). Phys Rev B: Condens Matter Mater Phys.

[6] Such B, Weiner D, Schirmeisen A, Fuchs H (2006). Appl Phys Lett.

[7] Braun D-A, Weiner D, Such B, Fuchs H, Schirmeisen A (2009). Nanotechnology.

[8] Gross L, Mohn F, Moll N, Liljeroth P, Meyer G (2009). Science.

[9] Mohn F, Gross L, Moll N, Meyer G (2012). Nat Nanotechnol.

[10] Baykara M Z, Schwendemann T C, Altman E I, Schwarz U D (2010). Adv Mater.

[11] Albrecht T R, Grütter P, Horne D, Rugar D (1991). J Appl Phys.

[12] Giessibl F J (2000). Appl Phys Lett.

[13] Langewisch G, Falter J, Fuchs H, Schirmeisen A (2013). Phys Rev Lett.

[14] Hölscher H, Langkat S M, Schwarz A, Wiesendanger R (2002). Appl Phys Lett.

[15] Sader J E, Jarvis S P (2004). Appl Phys Lett.

[16] Moll N, Gross L, Mohn F, Curioni A, Meyer G (2012). New J Phys.

[17] Langewisch G, Kamiński W, Braun D-A, Möller R, Fuchs H, Schirmeisen A, Pérez R (2012). Small.

[18] Baykara M Z, Dagdeviren O, Schwendemann T C, Mönig H, Altman E I, Schwarz U D (2012). Beilstein J Nanotechnol.

